# Large‐Scale Plasma Proteomics to Profile Pathways and Prognosis of Chronic Pain

**DOI:** 10.1002/advs.202410160

**Published:** 2025-03-06

**Authors:** Ze‐Yu Li, Qing Ma, Jie Zhang, Rui‐Ying Yin, Jia You, Qi‐Zheng Hao, Xin‐Rui Wu, Ju‐Jiao Kang, Lin‐Bo Wang, Yue‐Ting Deng, Yu‐Zhu Li, Chun Shen, Bang‐Sheng Wu, Jian‐Feng Feng, Yi‐Heng Tu, Xiao Xiao, Jin‐Tai Yu, Wei Cheng

**Affiliations:** ^1^ Institute of Science and Technology for Brain‐Inspired Intelligence Department of Neurology Huashan Hospital State Key Laboratory of Medical Neurobiology and MOE Frontiers Center for Brain Science Fudan University Shanghai 200433 China; ^2^ Key Laboratory of Computational Neuroscience and Brain‐Inspired Intelligence (Fudan University) Ministry of Education Shanghai 200433 China; ^3^ Key Laboratory of Brain Functional Genomics (MOE&STCSM), Affiliated Mental Health Center (ECNU), School of Psychology and Cognitive Science East China Normal University Shanghai 200062 China; ^4^ Department of Neurosurgery Huashan Hospital, Shanghai Medical College Fudan University Shanghai 200040 China; ^5^ National Center for Neurological Disorders Shanghai 200040 China; ^6^ Department of Computer Science University of Warwick Coventry CV4 7AL UK; ^7^ Fudan ISTBI–ZJNU Algorithm Centre for Brain‐inspired Intelligence Zhejiang Normal University Zhejiang 321004 China; ^8^ CAS Key Laboratory of Mental Health, Institute of Psychology Chinese Academy of Sciences Beijing 100101 China

**Keywords:** chronic pain, Mendelian randomization, plasma proteomic, prediction, UK Biobank

## Abstract

While increasing peripheral mechanisms related to chronic pain, the plasma proteomics profile associated with it and its prognosis remains elusive. This study utilizes 2923 plasma proteins and chronic pain of 51 644 participants from UK Biobank and finds 474 proteins linked to chronic pain in six sites: head, neck or shoulder, back, stomach or abdominal, hip, and knee, with 11 proteins sharing across pain sites. The identified proteins are largely enriched in immune and metabolic pathways and highly expressed in tissues like lungs and small intestines. Phenome‐wide analysis highlights the significance of pain‐related proteome on diverse facets of human health, and in‐depth Mendelian randomization validates 10 proteins (CD302, RARRES2, TNFRSF1B, BTN2A1, TNFRSF9, COL18A1, TNF, CD74, TNFRSF4, and BTN2A1) as markers of chronic pain. Furthermore, protein sets capable of classifying pain patients and healthy participants, particularly performing best in hip pain (area under curve, AUC = 0.725), are identified. Interestingly, the prediction of pain spreading over ten years achieves an AUC of 0.715, with leptin identified as a crucial predictor. This study delineates proteins associated with various pain conditions and identifies proteins capable of classifying pain and predicting pain spreading, offering benefits for both research and clinical practice.

## Graphical abstract

This study delineates both common and site‐specific proteins linked to various chronic pain conditions, elucidating their implications across molecular pathways and health status. These findings, particularly those proteins with genetic causal links to pain or predictive capabilities, have the potential to significantly benefit both research and clinical practice, further contributing to early risk assessment, pain management strategies, and therapeutic interventions.

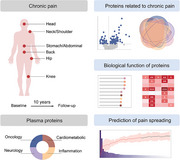

## 1 Introduction

Chronic pain constitutes a major component of global disability and disease burden,^[^ ^1^ ^]^ affecting ≈27.5% of the population worldwide,^[^ ^2^ ^]^ and reaching 27%–60% in individuals over 60 years old across healthcare surveys.^[^ ^3^, ^4^, ^5^ ^]^ Chronic pain, characterized by varying pain sites and changes in pain perception and tolerance^[^ ^6^ ^]^ during ageing process, often coexists with functional disabilities and increased risk of mortality in the elderly population.^[^ ^4^, ^7^ ^]^ Thus, the need for effective interventions is becoming increasingly pressing.^[^ ^8^ ^]^ Unfortunately, therapies for individuals suffering from pain are either inadequate for certain types of pain or cause intolerable side effects.^[^ ^9^ ^]^ This is mainly attributed to an incomplete understanding of the causes of pain occurrence and prognosis,^[^ ^10^ ^]^ as well as untimely screening and treatment.^[^ ^11^ ^]^ To address these challenges, a key strategy is the development of practical tools for early identification of high‐risk individuals in middle‐aged and elderly population, facilitating timely screening, diagnosis, treatment initiation, and improved outcomes. The risk factors for chronic pain primarily stem from the complex interplay between biological, psychological, and social abnormalities in the aging process, a concept acknowledged and recognized as the biopsychosocial model.^[^ ^12^ ^]^ On the biological side, however, it remains a challenge to develop quantitative and objective biomarkers with clinical utility that precede chronic pain for years and shape its prognosis.

Plasma proteomics shows promise in advancing this challenge.^[^ ^13^ ^]^ It offers insights into the complex interplay of genetic and environmental factors that contribute to disease by enabling the analysis of thousands of proteins simultaneously.^[^ ^14^ ^]^ Over the last decades, proteomics has been applied to establish diagnostic arrays^[^ ^15^ ^]^ for diseases including Alzheimer's^[^ ^16^ ^]^ and neuropsychiatric disorders.^[^ ^17^ ^]^ Furthermore, reductive medical translational models have shown that plasma proteins can effectively predict future at‐risk disorders.^[^ ^18^ ^]^ For instance, they can successfully predict the risk of future dementia in healthy adults,^[^ ^19^ ^]^ and only a few numbers of proteins can improve the identification of high‐risk individuals with diabetes.^[^ ^20^ ^]^ In the domain of pain research, however, the field is still in its infancy stage despite ongoing investigations into protein mechanisms^[^ ^21^, ^22^ ^]^ The majority of previous studies focused on comparative analyses, identifying differentially expressed proteins by comparing protein levels across various tissues (e.g., plasma, serum, saliva, cerebrospinal fluid) between individuals with chronic pain and healthy controls.^[^ ^23^, ^24^, ^25^ ^]^ From previous findings, immune‐inflammatory involvement is frequently implicated in chronic pain processing. For example, IL18R1, a receptor for the pro‐inflammatory protein IL18, correlated with pain visual analogue scale scores in children with juvenile idiopathic arthritis in a proteomic study.^[^ ^26^ ^]^ Relatively, less studies investigated pain‐related protein patterns correlate with clinical variables like pain intensity and psychological distress,^[^ ^27^, ^28^, ^29^ ^]^ and limited proteomic studies examined the effects of pain pharmacotherapy on protein profiles, which may also offer valuable insights into chronic pain mechanisms.^[^ ^30^, ^31^ ^]^

Past efforts to harness proteomics in pain research have encountered several limitations. First, previous studies focused on a narrow set of proteins,^[^ ^32^ ^]^ rather than utilizing proteomics to incorporate information from across the entire spectrum of plasma proteins, dealt with a small sample size,^[^ ^33^ ^]^ or focused solely on pain within a single tissue.^[^ ^34^, ^35^, ^36^ ^]^ This may result in candidate proteomic markers being challenging to reproduce in larger and more complex populations with diverse pain sites,^[^ ^22^ ^]^ as well as lacking commonality and specificity for various pain conditions. Second, although observational studies^[^ ^32^, ^37^ ^]^ have suggested an association between plasma proteins and pain, the causal effect of leveraging genetic variants of plasma proteins on chronic pain is currently unclear. This limits the potential for targeting proteins as novel therapeutic opportunities in translational development. Third, previous studies predominantly employed case‐control designs,^[^ ^38^ ^]^ leaving it uncertain whether plasma proteins can serve as clinical predictive biomarkers for the longitudinal prognosis of chronic pain in both pain‐free and pain‐afflicted populations.

Here, utilizing the UK Biobank Pharma Proteomics Project (UKB‐PPP), the largest targeted proteomic experiment to date, which encompasses data on 2923 proteins from 51644 middle‐aged and elderly participants, we systematically analyzed and established a comprehensive catalog of protein‐pain associations to address unanswered questions related to the risk proteomic signature of chronic pain and its prognostic indicators. We sought to characterize:^[^ ^1^ ^]^ shared and specific proteins linked to different pain conditions based on body site classifications;^[^ ^2^ ^]^ biological mechanisms of these pain‐related proteins;^[^ ^3^ ^]^ genetically determined effects of proteins on chronic pain;^[^ ^4^ ^]^ and which of the associated protein signatures are capable of classifying chronic pain and predicting pain spreading around a decade later.

## 2 Experimental Section

### 2.1 Study Population

This study used data from the UKB, a large cohort study that collected phenotypic and genetic data from around 500 000 individuals across the United Kingdom.^[^ ^39^ ^]^ The UKB cohort was approved by the North West Multi‐centre Research Ethics Committee (https://www.ukbiobank.ac.uk/learn‐more‐about‐uk‐biobank/about‐us/ethics), and all participants have provided written informed consent. This research has been conducted using the UK Biobank Resource under approved application number 19542. The main analysis in the study used participants’ demographic characteristics (Table S1, Supporting Information), chronic pain assessments (Tables S2 and S3, Supporting Information), and proteomic measurements (Table S4, Supporting Information). In addition, genotype data (Supplementary Method in the Supporting Information) was used for Mendelian randomization (MR), and blood indicators, lung function, neuropsychiatric diseases, digestive diseases, and brain volumes were used for phenome‐wide analysis (Supplementary Method in the Supporting Information).

Participants’ pain status included six body sites at baseline (2006–2010) and follow‐up (2019–2020): head, neck or shoulder, back, stomach or abdominal, hip, and knee. Participants were asked if they had experienced pain that interfered with their daily lives in the past month. If participants had pain and it lasted longer than three months, then they were judged to have chronic pain (Tables S2 and S3 and Supplementary Method, Supporting Information). First, we used pain status at baseline in the association analysis. For example, when identifying associations between headache and plasma proteins, we used participants with chronic headache as the case group and participants without any headache (chronic or acute) as the control group. The sample size for each chronic pain is shown in Table S2 (Supporting Information). Also, we used pain data at baseline to classify chronic pain and healthy participants. Participants with corresponding chronic pain were treated as the case group, and healthy participants (when answering questions in field 6159, the participants said they had not experienced any pain in the last month) were treated as the control group. In addition, we defined pain spreading as the number of pain sites at follow‐up minus the number at baseline, including all six body sites, to predict pain progress. The sample sizes and exclusion criteria used for each analysis are provided in Figure S1 (Supporting Information).

The UKB‐PPP cohort contained plasma samples from 54 219 participants, including 46 595 randomly selected participants, 6376 consortium‐chosen participants, and 1268 participants from a COVID‐19 repeat imaging study.^[^ ^40^, ^41^, ^42^ ^]^ For each participant, a total of 2923 unique proteins were obtained from UKB, which were measured across eight protein panels (cardiometabolic, cardiometabolic II, inflammation, inflammation II, neurology, neurology II, oncology, and oncology II). The protein levels were provided by preprocessing them into Normalized Protein eXpression (NPX) values (Supplementary Method in the Supporting Information). More details about proteins and corresponding panels are provided in Table S4 (Supporting Information).

### 2.2 Statistical Analysis

#### 2.2.1 Association Analysis between Chronic Pain and Plasma Proteins

To explore the relationship between chronic pain at six body sites and 2923 plasma proteins, we performed an association analysis. We used the logistic regression model and controlled for the necessary covariates. Covariates included demographic characteristics such as age, sex, BMI, race (White or non‐White), Townsend index, and highest educational qualification (Table S1, Supporting Information), and technical parameters such as assessment center, batch, and sample age (time between blood sampling and protein measurement). The significance threshold was defined as *P* < 2.85 × 10^−6^ (Bonferroni correction).

#### 2.2.2 Pathway Enrichment and Tissue Expression Analysis

To explore the underlying biological mechanisms of pain‐related proteins, we analyzed pathway enrichment and tissue expression of significant proteins related to six pain sites and their combinations. Pathway enrichment analyses were conducted by R package clusterProfiler,^[^ ^43^, ^44^ ^]^ and gene set databases included Gene Ontology (GO) and Kyoto Encyclopedia of Genes and Genomes (KEGG). The GO terms were divided into three categories: Biological Process (BP), Cellular Component (CC), and Molecular Function (MF). The tissue‐specific type expression analyses were performed by the GENE2FUNC implemented in the Functional Mapping and Annotation (FUMA).^[^ ^45^ ^]^ The tissue analyses used the GTEx v8^[^ ^46^ ^]^ database and contained 54 tissue types.

#### 2.2.3 Mendelian Randomization

We performed the two‐sample MR analysis using inverse variance weighted (IVW), MR Egger, weighted median, simple mode, and weighted mode methods, implemented in the R package TwoSampleMR.^[^ ^47^, ^48^ ^]^ We used the protein as the exposure and the chronic pain as the outcome. The genome‐wide association study (GWAS) summary data of proteins were from the previous study,^[^ ^42^ ^]^ and the GWAS of chronic pain were calculated by ourselves, excluding participants with protein data. The GWAS was performed using the software PLINK 2.0^[^ ^49^ ^]^ with age, sex, and the top 20 principal components (PCs) used as covariates. In the MR analysis, we selected SNPs with *P* < 5 × 10^−8^ as instrumental variables (IVs) and removed correlated SNPs (*r* ^2^ > 0.1). When the number of IVs was less than five, we relaxed the SNP threshold to 1 × 10^−6^. In addition, we performed the heterogeneity test using IVW and MR Egger methods and the pleiotropy test using the MR Egger method.

#### 2.2.4 Classifying Chronic Pain and Predicting Pain Spreading

We developed pain risk models using the Light Gradient Boosting Machine (LightGBM)^[^ ^50^ ^]^ algorithm to investigate the classification and prediction capabilities of plasma proteins. For pain classification, the model aimed to determine whether the participants had chronic pain at one body site (class 1) or were healthy (without any pain, class 0) at baseline. For pain spreading, the model predicted whether the number of pains would increase (class 1), remain the same (class 0), or decrease (class −1) during the follow‐up. We randomly divided the training set and the test set according to two‐thirds and one‐third. Model training, feature selection, and parameter tuning were conducted exclusively in the training set, while the test set, which was entirely excluded from these processes, was used solely to verify the model's generalization.

In the training set, we used tenfold cross‐validation to perform features and hyperparameters selection. First, 2923 proteins were used as model inputs to measure the importance of proteins according to the information gain of the LightGBM model. Next, we selected the top 100 important proteins and demographic characteristics such as age, sex, BMI, race, and Townsend index and performed hyperparameter tuning by randomly sampling 200 candidate parameter sets from a predefined hyperparameter grid using a random search strategy. The parameter set with the highest area under curve (AUC) value was selected as the final parameter set. Finally, we verify the performance of the model with an independent test set. The top 100 proteins and demographic characteristics selected by the training set were used as input features, and the optimal parameters generated from the training set were used.

## 3 Results

### 3.1 Description of Study Population and Data

**Figure 1:**
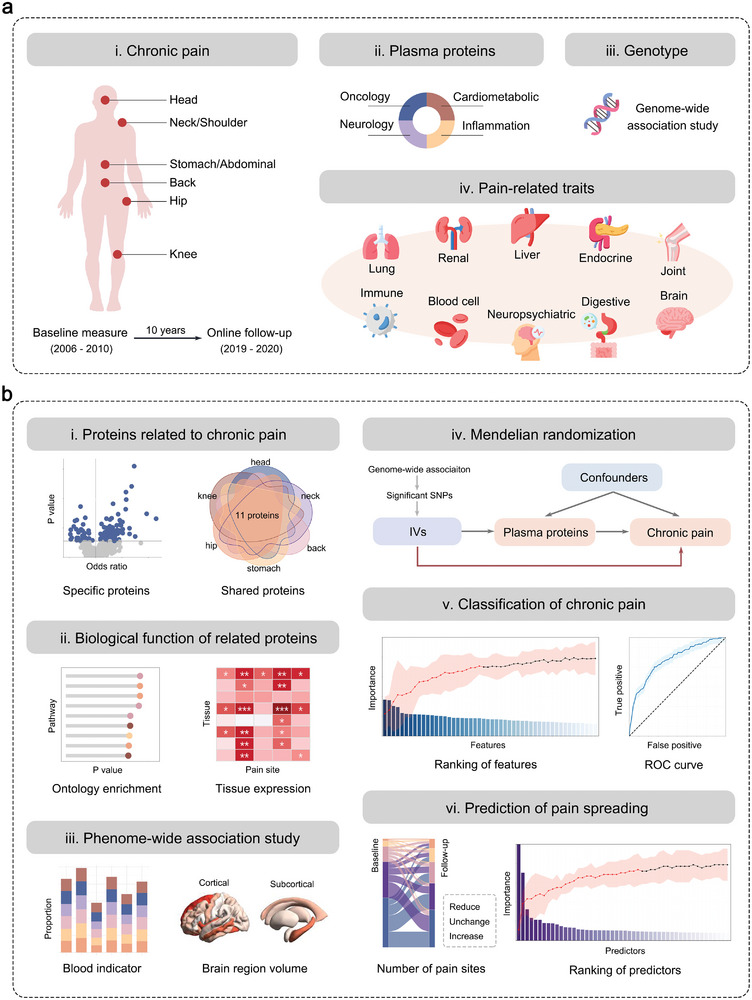
The overall study design. a) Data used in the study, including chronic pain, plasma proteins, genotype data, and pain‐related traits. i) Chronic pain was measured at baseline and online follow‐up, including six body sites: head, neck or shoulder, back, stomach or abdominal, hip, and knee. ii) Plasma proteins were divided into four categories: cardiometabolic, inflammation, neurology, and oncology. iii) Genotype data were used in the genome‐wide association analysis. iv) Pain‐related phenotypes included blood indicators (liver function, renal function, endocrine, immune, joint, and blood cell), lung function, neuropsychiatric diseases, digestive diseases, and brain volumes. b) Analysis process in the study. i) Association analysis between chronic pain and plasma proteins. ii) Biological function analysis of pain‐related proteins, including ontology pathway and tissue expression analyses. iii) Phenome‐wide association analysis of pain‐related proteins. iv) Mendelian randomization of chronic pain and proteins. v) Classification of chronic pain at baseline. vi) Prediction of pain spreading after a ten‐year follow‐up.

We included 51 644 individuals with both blood plasma protein data and pain questionnaire at baseline from the UK Biobank (UKB), aged from 39 to 70 at enrollment, comprising 54% women and 94% white individuals (Table S1, Supporting Information). Chronic pain across six body sites were involved, including head, neck or shoulder, back, stomach or abdominal, hip and knee. Individuals’ pain was measured through the questionnaire at baseline (2006–2010, Table S2, Supporting Information) and online follow‐up (2019–2020, Table S3, Supporting Information). Plasma samples were processed using the Olink Explore 3072, and 2923 unique proteins were obtained (Table S4, Supporting Information). The overall study design is shown in **Figure** **1**.

### 3.2 Association between Chronic Pain and Targeted Plasma Proteomics

**Figure 2:**
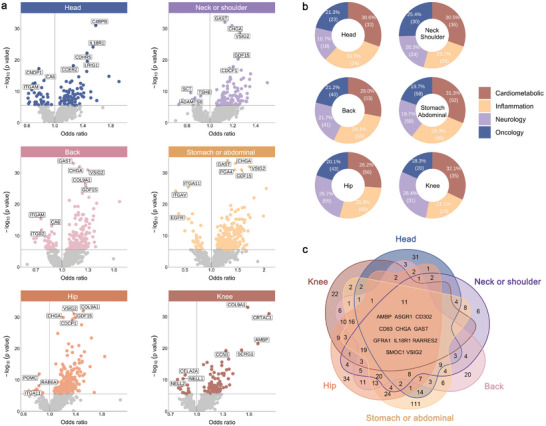
Relationship between chronic pain and plasma proteins. a) Scatter plots show the associations between 2923 proteins and chronic pain at six body sites. Logistic regression model was used to examine the association between protein levels and chronic pain. Analyses were adjusted for age, sex, BMI, race, Townsend deprivation index, highest educational qualification, assessment center, batch, and sample age. *P*‐values shown are two‐sided and not adjusted for multiple comparisons. The gray horizontal line indicates the significance threshold (*P* < 2.85 × 10^−6^), and the colored dots above the line indicate that the protein was significantly associated with the disease. The vertical line represents a dividing line with an odds ratio of 1, and the point to the right of the line indicates that the higher level of this protein increased the risk of disease. b) Plots show the proportion of each category of proteins significantly associated with pain, including four categories: cardiometabolic, inflammation, neurology, and oncology. c) Venn diagram shows the overlap of proteins significantly associated with pain at different body sites.

We identified 1032 significant associations between proteins and six pain sites following Bonferroni correction (*P* < 2.85 × 10^−6^), involving a total of 474 unique proteins (**Figure** **2**a and Table S5, Supporting Information). Among these associations, 108 proteins were associated with headache, of which C4BPB (odds ratio [OR]  =  1.59, *P* = 1.67 × 10^−39^), IL18R1 (OR = 1.55, *P* = 7.36 × 10^−25^), and CDHR5 (OR = 1.46, *P* = 6.31 × 10^−23^) had the strongest associations. Neck or shoulder pain, back pain, and stomach or abdominal pain had 118, 189, and 294 proteins associated with them, respectively. The first three proteins were the same for all three pains, namely GAST, CHGA, and VSIG2, of which GAST was the most significant (neck or shoulder, OR = 1.13, *P* = 5.31 × 10^−41^; back, OR = 1.13, *P* = 1.11 × 10^−44^; stomach or abdominal, OR = 1.31, *P* = 2.79 × 10^−83^). For hip pain and knee pain, 214 and 109 proteins were associated, respectively, and COL9A1 had the most significant associations (hip, OR = 1.52, *P* = 3.76 × 10^−49^; knee, OR = 1.59, *P* = 7.31 × 10^−93^).

Then, we examined the proportion of proteins significantly associated with pain in six body sites, among which the proportions of inflammation in headache (31.5%), back pain (29.1%), and hip pain (28.0%) were the highest, while the proportions of cardiometabolic in others (neck or shoulder, 30.5%; stomach or abdominal, 31.3%; knee, 32.1%) were the highest (Figure 2b). Furthermore, we assessed protein overlap across various pain sites and found 11 shared proteins for each pain type, including AMBP, ASGR1, CD302, CD83, CHGA, GAST, GFRA1, IL18R1, RARRES2, SMOC1, and VSIG2 (Figure 2c and Table S6, Supporting Information).

### 3.3 Biological Function of Pain‐Related Proteins

**Figure 3:**
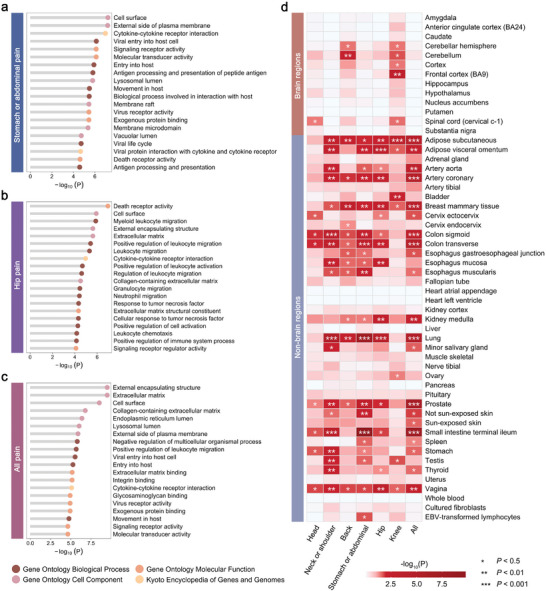
Biological function of pain‐related proteins. a) Plot shows the pathway enrichment of proteins related to stomach or abdominal pain. Analyses were conducted by R package clusterProfiler, and gene set databases included Gene Ontology and Kyoto Encyclopedia of Genes and Genomes. The GO terms were divided into three categories: Biological Process, Cellular Component, and Molecular Function. *P*‐values shown are two‐sided and not adjusted for multiple comparisons. b) Plot shows the pathway enrichment of proteins related to hip pain. c) Plot shows the pathway enrichment of all 474 pain‐related proteins. d) Plot shows the tissue‐specific type expression of pain‐related proteins. Analyses were performed by the GENE2FUNC implemented in Functional Mapping and Annotation (FUMA). The tissue analyses used the GTEx v8 database and contained 54 tissue types. *P*‐values shown are two‐sided and not adjusted for multiple comparisons.

Next, we examined the biological pathways involved in these pain‐related proteins at different pain sites (Tables S7–S12, Supporting Information). For example, proteins associated with stomach or abdominal pain were enriched in several immune‐related processes such as cytokine–cytokine receptor interaction (*P* = 1.07 × 10^−7^) and viral entry into host cell (*P* = 7.38 × 10^−7^) (**Figure** **3**a). Proteins associated with hip pain were enriched in several leukocyte‐related processes such as myeloid leukocyte migration (*P* = 1.33 × 10^−6^) and positive regulation of leukocyte migration (*P* = 3.89 × 10^−6^) (Figure 3b). We also examined the pathways linked to all 474 proteins associated with pain across six sites. We found that cellular components such as external encapsulating structure (*P* = 3.23 × 10^−10^), and pathways such as negative multicellular organismal process regulation (*P* = 1.60 × 10^−6^) and positive leukocyte migration regulation (*P* = 2.14 × 10^−6^), were enriched for these pain‐related proteins (Figure 3c and Table S13, Supporting Information).

We also characterized the expression profiles of these proteins in 54 tissue types using GTEx v8.^[^ ^46^ ^]^ The small intestine terminal ileum showed the most significant enrichment for proteins linked to neck or shoulder pain (*P* = 2.21 × 10^−4^), stomach or abdominal pain (*P* = 1.61 × 10^−10^), and pain across all six sites (*P* = 2.27 × 10^−8^). In addition, significant enrichments were found in lung tissue, including proteins associated with pain involving neck or shoulder (*P* = 6.65 × 10^−6^), stomach or abdominal (*P* = 3.33 × 10^−8^), hip (*P* = 1.05 × 10^−4^), and all body sites (*P* = 7.75 × 10^−6^) (Figure 3d and Table S14, Supporting Information).

### 3.4 Phenome‐Wide Association Analysis of Pain‐Related Proteins

**Figure 4:**
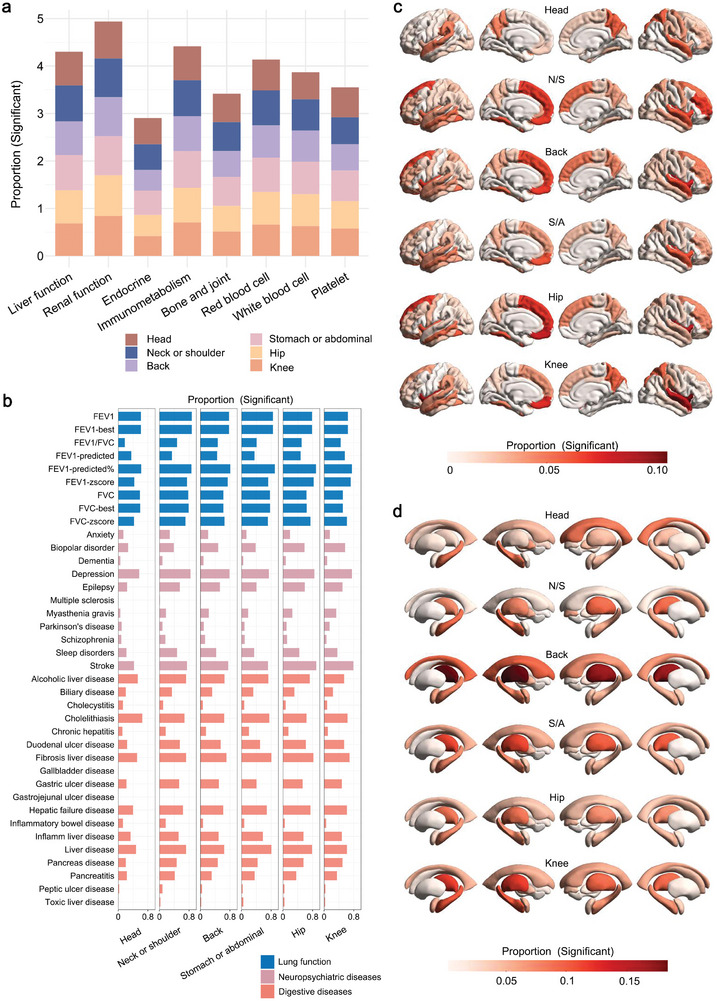
Phenome‐wide association of pain‐related proteins. Phenome‐wide association used linear and logistic regression models to examine associations between protein levels and phenotypes. Analyses were adjusted for age, sex, BMI, race, Townsend deprivation index, highest educational qualification, assessment center, batch, sample age, and total intracranial volume (only used in the brain volume analysis). a) Proportion of pain‐related proteins significantly associated with each blood indicator category. b) Proportion of pain‐related proteins significantly associated with each phenotype in lung function, neuropsychiatric diseases, and digestive diseases. c,d) Proportion of pain‐related proteins significantly associated with each cortical and subcortical region volume. N/S, neck or shoulder; S/A, stomach or abdominal.

To better understand the underlying mechanisms of pain‐related proteins, we selected the phenotypes that were associated with chronic pain and reflected human health, which may help us understand pathways contributing to chronic pain. We then investigated evidence from cross‐omics to examine phenome‐wide associations, encompassing phenotypes from blood indicators, lung function, neuropsychiatric diseases, digestive diseases, and brain volumes. For blood indicators, lung function and diseases analysis, the Bonferroni correction was used to test multiple corrections. FDR correction was used for brain volumes analysis. Strong associations between pain‐related proteins and blood indicators were predominantly concentrated in markers of liver function, renal function, and immunometabolism (**Figure** **4**a; Tables S15 and S16, Supporting Information), with cystatin C (CysC) and alkaline phosphatase (ALP) representing the highest associations for almost all pain conditions (Table S17, Supporting Information). In terms of common health issues, pain‐related proteins showed significant correlations with lung function, neuropsychiatric diseases, and digestive diseases. Notably, leptin (LEP), GDF15, and ADM showed the strongest associations with lung function, while BST2, THBS2, and CEACAM1 exhibited the most significant associations with digestive diseases (Tables S18 and S19, Supporting Information). In neuropsychiatric diseases, depression and stroke represented the strongest associations with proteins across nearly every pain condition (Figure 4b and Table S20, Supporting Information). Also, we identified significant associations between pain‐related proteins and brain volumes. PTPRN2 exhibited associated with numerous cortical volumes, with particularly significant brain regions including the rostral middle frontal, precuneus, lateral occipital, inferior temporal, cuneus, and superior parietal areas (Table S21, Supporting Information). Among associations, insula and medial orbitofrontal cortex showed the highest correlations across almost all pain conditions (Figure 4c and Table S22, Supporting Information). BCAN was also linked to various cortical and subcortical brain volumes, with the most significant brain regions including rostral middle frontal, superior temporal, hippocampus, lateral ventricle, pallidum, and thalamus. In addition, MZB1 and GDF15 were significantly associated with the hippocampus, while GDF15 and PLAUR were linked to the thalamus (Figure 4d; Tables S23 and S24, Supporting Information).

### 3.5 Genetic Effect of Proteins on Chronic Pain by Mendelian Randomization Analysis

**Figure 5:**
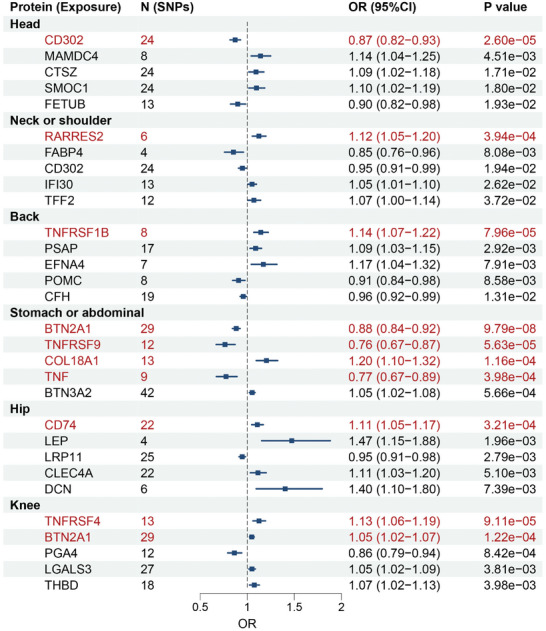
Mendelian randomization (MR) analysis between chronic pain and proteins. Plot shows the top five associated proteins for pain in each body site in the MR analysis using the inverse variance weighted (IVW) method. Two‐sample MR analysis was performed using R package TwoSampleMR, with the protein as the exposure and chronic pain as the outcome. *P*‐values shown are two‐sided and not adjusted for multiple comparisons. The red font indicates that the association remained significant after FDR correction (*P*_FDR_ < 0.05).

We performed MR analysis on the significant proteins identified in the association analysis, using the protein as the exposure and chronic pain as the outcome. In total, we identified 95 genetic associations (*P* < 0.05) between proteins and chronic pain using the inverse variance weighted method (Table S25, Supporting Information), of which 10 associations exhibited a more significant relationship following multiple comparisons (*P*_correction_ < 0.05, FDR correction). Among them, the top five significant associations in each pain are shown in **Figure** **5**. Genetically, CD302 was associated with headache (*P* = 2.60 × 10^−5^), RARRES2 was associated with neck or shoulder pain (*P* = 3.94 × 10^−4^), TNFRSF1B was associated with back pain (*P* = 7.96 × 10^−5^), and CD74 was associated with hip pain (*P* = 3.21 × 10^−4^). The plasma levels of BTN2A1 (*P* = 9.79 × 10^−8^), TNFRSF9 (*P* = 5.63 × 10^−5^), COL18A1 (*P* = 1.16 × 10^−4^), and TNF (*P* = 3.98 × 10^−4^) were associated with stomach or abdominal pain, while BTN2A1 was also associated with knee pain (*P* = 1.22 × 10^−4^). In addition, TNFRSF4 correlated with knee pain (*P* = 9.11 × 10^−5^). For details on the 84 proteins not surviving multiple comparison correction but genetically associated with different pain conditions, refer to Table S25 (Supporting Information). The results of the heterogeneity test and the pleiotropy test are given in Tables S26 and S27 (Supporting Information). Meanwhile, the associations across other MR methods were examined, including MR Egger, weighted median, simple mode, and weighted mode (Table S28, Supporting Information).

### 3.6 Classifying Pain Patients and Healthy Participants

**Figure 6:**
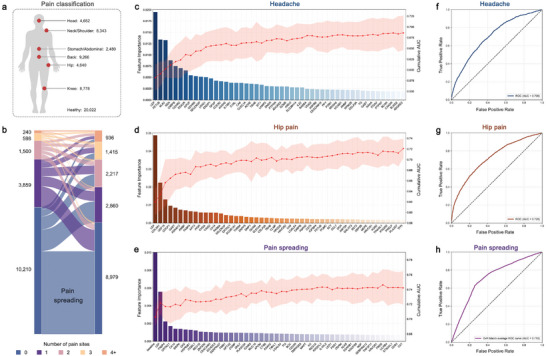
Classification and prediction of chronic pain. a) Plot shows the sample sizes of chronic pain patients and healthy individuals at baseline. b) Plot shows changes in the number of pain sites between baseline and follow‐up. c) The importance of features in the headache classification model. Bar chart shows the ranking of the importance of the variables according to their contribution to the model classification. Line chart shows the cumulative AUC value of the model that adds a feature in order at each iteration. d) The importance of features in the hip pain model. e) The importance of features in the pain spreading model. f) The receiver operating characteristic (ROC) curve of the headache model. In the coordinate system, the vertical axis is the true positive rate, and the maximum value is 1. The horizontal axis is the false positive rate, and the maximum value is 1. g) ROC curve of the hip pain model. h, ROC curve of the pain spreading model using the one‐versus‐rest (OVR) macro‐average method.

We used the levels of 2923 proteins to classify pain patients and healthy participants at baseline, and the number of individuals with chronic pain is shown in **Figure** **6**a. We selected 100 candidate proteins with higher predictive weights through the machine learning (ML) model, many of which were previously associated with chronic pain. To visualize as many proteins as possible, we showcase the top 50 proteins based on their significance in prediction performance (Figure 6c,d, and Figures S2–S5, Supporting Information). Subsequently, we used 100 proteins and demographic characteristics as model inputs and selected the optimal parameters through cross‐validation. Finally, we validated the trained model on the test set. Among them, the model classification of hip pain and healthy participants had the best performance, with an AUC value of 0.725 (Figure 6g), followed by stomach or abdominal pain (AUC = 0.712, Figure S8, Supporting Information), headache (AUC = 0.706, Figure 6f), knee pain (AUC = 0.701, Figure S9, Supporting Information), back pain (AUC = 0.673, Figure S7, Supporting Information), and neck or shoulder pain (AUC = 0.650, Figure S6, Supporting Information).

### 3.7 Predicting Spread of Chronic Pain in Aging Process

We implemented a three‐category model to predict changes in number of pain sites about 10 years later: decrease, no changes, or increase (Figure 6b). Using the same method as described above, we ultimately identified 100 significant proteins which exhibited the best performance with an AUC value of 0.715 (Figure 6h). Likewise, in Figure 6e, we presented the top 50 proteins with the higher predictive weight. Notably, the number of pain sites at baseline showed the greatest predictive weight, followed by the level of LEP.

## 4 Discussion

In this study, we employed the most comprehensive proteomic data to date from the UK Biobank, to systematically investigate how targeted plasma proteomics contribute to understanding the molecular mechanisms of chronic pain and its prognosis in clinical settings. Out of 2923 proteins, we identified 474 proteins associated with pain, with 11 shared across six body sites, alongside unique proteins specific to each site. Subsequently, by leveraging phenotypic data that nearly comprehensively describes an individual's health, we uncovered consistent and significant manifestations of pain‐related proteins in various aspects of human health, including blood indicators, lung function, neuropsychiatric diseases, digestive diseases, and brain volumes across all pain sites. In‐depth Mendelian randomization analysis captured genetic information from both proteins and pain, identifying 10 significant proteins with a potential causal effect on chronic pain. Final predictive analysis enriched our understanding by revealing that specific proteins have the capacity to predict pain spreading over a ten‐year period, with some implicated in genetic causation. These findings serve to fill a critical gap in our understanding, highlighting proteomic biomarkers that offer new insights into the pathology of chronic pain, its occurrence, progression, and implications for clinical intervention.

Our findings underscore the value of targeted plasma proteomics in combination with a large‐scale population cohort to unravel the complex mechanisms of chronic pain. This is particularly significant as proteomic research in the field of chronic pain remains largely exploratory.^[^ ^21^ ^]^ We found 11 proteins shared by variant pain sites, suggesting that different chronic pain conditions may involve multiple common biological pathways, including immune‐inflammatory responses, neural system functions, metabolic regulation, and tissue development and repair. Reflecting a neuroimmune perspective on pain,^[^ ^51^ ^]^ IL18R1, CD83, CD302, and AMBP are pivotal in engaging the immune system, mediating immune‐inflammatory interactions, and facilitating tissue repair, which potentially influences the development of pain by activating immune cells and releasing inflammatory molecules.^[^ ^26^, ^52^, ^53^, ^54^ ^]^ Although critical role of immunity‐inflammation in chronic pain mechanisms, few previous studies have implicated the involvement of these specific proteins in the processing of chronic pain, with the exception of IL18R1. IL18R1, a receptor for the pro‐inflammatory protein IL18, was found to correlate with the pain visual analogue scale score in children with juvenile idiopathic arthritis in a proteomic study.^[^ ^26^ ^]^ In addition, an animal study demonstrated that IL18 mediates central sensitization in the spinal cord by connecting microglial P2Y12‐SFK‐p38 signaling, contributing to cisplatin‐induced pain hypersensitivity.^[^ ^55^ ^]^ Similarity, GFRA1, belonging to GDNF (glial cell line‐derived neurotrophic factor) family, is implicated in the neural growth and survival that are crucial for modulating the immune response, directly linking neural dysfunction to neuropathic pain and pain following nerve or tissue injury.^[^ ^56^, ^57^ ^]^ Studies have shown that GDNF regulates pain responses by modulating sensory neuron sensitization to stimuli,^[^ ^58^, ^59^ ^]^ such as preventing and reversing sensory abnormalities in neuropathic pain animal models.^[^ ^60^ ^]^ It is also considered a promising therapeutic target for neuropathic pain and addiction.^[^ ^57^ ^]^

Moreover, our findings extend to proteins not typically associated with immunity or inflammation but strongly associated with chronic pain. For instance, Gastrin (GAST) and Chromogranin A (CHGA), traditionally linked to gastrointestinal functions and disorders,^[^ ^61^, ^62^ ^]^ hint at a potential connection between digestive processes and chronic pain across various body sites. In addition, some of the shared proteins we identified, like ASGR1 and SMOC1, though not directly linked to pain, serve as biomarkers for disease diagnosis and treatment, highlighting their importance in the broader context of pain pathology. ASGR1, primarily found in myeloid cells and the liver, is an emerging therapeutic target for hypercholesterolemia due to its role in decreasing lipid levels.^[^ ^63^, ^64^, ^65^ ^]^ SMOC1, part of the SPARC (secreted protein acidic and rich in cysteine) family, plays diverse roles in tissue development and cell–matrix interactions^[^ ^66^ ^]^ and is often implicated in Alzheimer's disease,^[^ ^67^ ^]^ further indicating its potential involvement in chronic pain progression. It is important to note that there is currently no evidence supporting the involvement of these proteins in pain processing. Nevertheless, they show promise as early biomarkers for chronic pain that may develop years later, representing a novel perspective distinct from existing study frameworks and underscoring the need for further investigation into their potential role in pain modulation.

This study is the first to systematically evaluate the genetic causal link of plasma proteomics to chronic pain, underscoring the pivotal roles of the immune response in the different types of pain and their progressions.^[^ ^68^ ^]^ We have identified two main types of genetically determined proteins that significantly influence both inflammatory pain and nociplastic pain: the tumor necrosis factor receptor superfamily (TNFRSF) and the cluster of differentiation (CD) protocol. These findings suggest that the immune system's response, through TNFRSF members (TNFRSF1B for back pain; TNFRSF9 for stomach or abdominal pain; TNFRSF4 for knee pain) and CD molecules, plays a crucial role in pain initiation, prognosis, and progression. Specifically, tumor necrosis factors (TNFs), through an extracellular cysteine‐rich domain, genetically influence various pain conditions. TNFRSF4, known as OX40 (CD134), a co‐stimulatory molecule important for T‐cell function, exhibits elevated levels in conditions associated with immune response to neural damage.^[^ ^69^, ^70^ ^]^ This indicates that the protein‐related immune system's reaction to nerve injury contributes to the development and maintenance of nociplastic pain.^[^ ^71^ ^]^ Moreover, we have identified CD molecules like CD302 and CD74 in the study. CD302, which we found to be genetically associated with head, neck, and shoulder pain, emerges as a critical marker across all pain types. CD74, also known as the MHC class II invariant chain, had been associated in patients with spondyloarthritis,^[^ ^72^ ^]^ back pain,^[^ ^73^, ^74^ ^]^ and bladder pain ^[^ ^75^ ^]^ indicating a shared immunological foundation underlying different forms of inflammatory pain. The findings that both nociplastic and inflammatory pain conditions involve these proteins underscore the complex interplay between the immune system and pain. It suggests that pain, whether originating from nerve damage (nociplastic) or inflammation (inflammatory), shares common biological underpinnings that could be addressed through targeted therapeutic interventions.

Our results highlight the effectiveness of blood protein biomarkers in classifying chronic pain and predicting the progression of pain, with a special emphasis on leptin (LEP). LEP, an appetite‐regulatory hormone and proinflammatory adipokine,^[^ ^76^ ^]^ emerges as a key biomarker not only for predicting pain spreading but also for its potential genetic causal relationship with hip pain as revealed by Mendelian randomization analysis. This discovery builds on the earlier observation in 2016,^[^ ^77^ ^]^ that elevated levels of LEP in women's blood were associated with increased self‐reported physical pain, marking the first connection of LEP to pain experiences. More recently, a cross‐sectional study found that LEP indicators were associated with chronic widespread pain in individuals with knee pain.^[^ ^78^ ^]^ Utilizing a longitudinal study design, our study confirms the role of LEP in pain spreading, which has not been discovered in previous pain studies, and also updates the understanding of LEP ’s function. However, in six pain classification models, the performance for neck or shoulder pain (AUC = 0.65) and back pain (AUC = 0.673) was relatively low compared to other pain sites. We thought it may be attributed to the multifactorial nature of these pain,^[^ ^79^, ^80^, ^81^ ^]^ limited representation of relevant features in the dataset, or imbalanced data distribution.^[^ ^82^ ^]^ In addition, subjective variability in pain reporting among individuals could contribute to the model's challenges in classification.^[^ ^83^ ^]^ Future research should consider incorporating additional psychological and contextual variables or applying data balancing techniques to improve the model's performance.

This study systematically elucidates the extensive impact of pain‐related proteins on various organic functions and diseases, underscoring the intricate relationships between pain and comorbid conditions. Notably, the association between pain‐related proteins and lung function is highlighted, corroborating previous studies that lung function coexists with neck pain^[^ ^84^ ^]^ and that improved lung function can help relieve pain.^[^ ^85^ ^]^ Furthermore, our findings reveal significant correlations between pain‐related proteins and brain volume alterations, particularly in regions responsible for executive, emotional, and spatial processing,^[^ ^86^ ^]^ suggesting a profound connection between chronic pain and cognitive as well as affective disorders.^[^ ^87^, ^88^, ^89^ ^]^ More interestingly, the distribution of proteins associated with brain volume in headache patients differed slightly from that in other pain conditions, with a focus on the precuneus, superior parietal, and hippocampus regions, while the latter concentrated on the insula, medial orbitofrontal, and thalamus regions. This indicates that nociplastic pain may involve distinct brain regions and peripheral molecular mechanisms compared to inflammation pain. However, our study did not distinguish between primary and secondary pain on the basis of considering comorbidities, and future studies need to further explore the different biological mechanisms of primary and secondary pain.

This study possesses several strengths. Most noteworthy, our study revealed the biological basis behind chronic pain through the quantification of objective biological measures, reducing the reliance on self‐reported pain experiences that may vary with psychological and cultural factors. Currently, protein research in chronic pain is in its infancy stage.^[^ ^21^ ^]^ Our study addresses this gap by employing the most comprehensive proteome to date through systematic research. Unlike many pain studies that concentrate on cross‐sectional data from a single pain site, our research utilizes a longitudinal sample across multiple pain sites to expand our understanding of the role proteomes play in pain. Nonetheless, several limitations should be noted. First, UKB samples are generally biased toward a healthier population, and the occurrence of individuals with pain may be lower compared to the broader UK population or other reported cohorts. Pain data in the UKB are based on self‐reported and lack validated pain questionnaires. However, the substantial scale of this cohort enhances the scientific reliability of inferences regarding proteome‐wide links with pain. Second, while Olink assays offer extensive proteome coverage, their capacity is still confined to quantifying a finite set of proteins. This limitation may result in the omission of numerous interesting proteins not represented on the panel or those undergoing post‐translational modifications, in stark contrast to the unbiased nature inherent in mass spectrometry. However, current proteomics are most extensive, especially with massive population scales. Third, due to data limitations, we were unable to comprehensively consider pain‐related comorbidities and underlying mechanisms. The International Association for the Study of Pain (IASP) classifies pain into nociceptive, neuropathic, and nociplastic pain.^[^ ^1^ ^]^ However, we must clarify that distinguishing between these three categories can be challenging due to considerable overlap and the prevalence of mixed pain states.^[^ ^1^, ^90^ ^]^ Future studies should include more comprehensive information to distinguish pain categories based on mechanisms.

In conclusion, our study unveiled biomolecular markers indicative of chronic pain through the objective quantification of plasma proteins in a cohort of 51644 individuals. Our findings delineate both common and site‐specific proteins linked to various chronic pain conditions, elucidating their implications across molecular pathways, health status, and diseases. Moreover, we identified proteins that signal the potential spread of chronic pain. These findings, particularly those proteins with genetic causal links to pain or predictive capabilities, have the potential to significantly benefit both research and clinical practice, further contributing to early risk assessment, pain management strategies, drug development, and therapeutic interventions.

## Conflict of Interest

The authors declare no conflict of interest.

## Author Contributions

Z.‐Y.L., Q.M., J.Z., and R.‐Y.Y. contributed equally to this work. Conceptualization: W.C., J.‐T.Y., X.X.; Methodology: Z.‐Y.L., Q.M., R.‐Y.Y., J.Y., J.‐J.K., L.‐B.W.; Investigation: Z.‐Y.L., Q.M., J.Z., Y.‐T.D., Y.‐Z.L., C.S.; Visualization: Z.‐Y.L., Q.‐Z.H., W.Z., B.‐S.W.; Funding acquisition: W.C., J.‐T.Y., J.‐F.F.; Project administration: W.C., J.‐T.Y.; Supervision: W.C., J.‐F.F.; Writing – original draft: Z.‐Y.L., Q.M., R.‐Y.Y., J.Y., X.‐R.W.; Writing – review & editing: Z.‐Y.L., Q.M., J.Z., X.X., Y.‐H.T.

## Supporting information



Supporting Information

Supporting Information

## Data Availability

The data that support the findings of this study are openly available by application in UK Biobank at https://www.ukbiobank.ac.uk/.
