# Supplementary material for: Large‐Scale Plasma Proteomics to Profile Pathways and Prognosis of Chronic Pain
Source: Adv Sci (Weinh). 2025 Mar 6;12(16):2410160. doi: 10.1002/advs.202410160 (PMC12021123; doi:10.1002/advs.202410160)
Supplement: Supplementary file 1 — Supporting Information [file ADVS-12-2410160-s001.docx]

**Supplementary Material**

**Large-scale plasma proteomics to profile pathways and prognosis of chronic pain**

**Content**

[Supplementary Methods 1](#_Toc189642037)

[Assessment of chronic pain 1](#_Toc189642038)

[Measurement of plasma proteins 1](#_Toc189642039)

[Genotype data 2](#_Toc189642040)

[Phenome-wide association analysis of pain-related proteins 2](#_Toc189642041)

[References 4](#_Toc189642042)

[Supplementary Figures 5](#_Toc189642043)

[Figure S1. The sample sizes and exclusion criteria used for analyses 5](#_Toc189642044)

[Figure S2. The importance of features in the neck or shoulder pain model 6](#_Toc189642045)

[Figure S3. The importance of features in the back pain model 7](#_Toc189642046)

[Figure S4. The importance of features in the stomach or abdominal pain model 8](#_Toc189642047)

[Figure S5. The importance of features in the knee pain model 9](#_Toc189642048)

[Figure S6. ROC curve of the neck or shoulder pain model 10](#_Toc189642049)

[Figure S7. ROC curve of the back pain model 11](#_Toc189642050)

[Figure S8. ROC curve of the stomach or abdominal pain model 12](#_Toc189642051)

[Figure S9. ROC curve of the knee pain model 13](#_Toc189642052)

# Supplementary Methods

**Assessment of chronic pain**

In the main analysis of the association between chronic pain and proteins, we used participants' pain status in six body sites at baseline (2006-2010), including head, neck or shoulder, back, stomach or abdominal, hip, and knee. Participants were asked if they had experienced pain that interfered with their daily lives in the past month. If participants had pain and it lasted longer than three months, they were judged to have chronic pain (**Table S2**). In the prediction of pain spreading, pain status at online follow-up (2019-2020) was used. In the questionnaire, participants were asked if they had been suffering from pain or discomfort on or off for more than 3 months. If they said yes, we determined that they were suffering from chronic pain. The questionnaire went on to ask about the body site and the extent of the pain (**Table S3**). Pain spreading was defined by the number of pain sites at follow-up minus the number at baseline, including all six body sites.

**Measurement of plasma proteins**

Blood samples were collected at participants’ first visits to the recruitment center during their baseline assessments. Samples were collected in EDTA (9 ml) tubes and fractioned into 850 μL aliquots to isolate plasma, buffy coat, and red cells. The plasma samples were stored at -80°C before being transferred on dry ice to the Olink Analysis Service in Sweden. About 50,000 participants sampled from random baseline, consortium selected, COVD-10 imaging, or a combination were selected, and their plasma samples were parallelly quantified using the antibody-based Olink Explore™ Proximity Extension Assay between April 2021 and February 2022. For each participant, a total of 2,923 unique proteins were measured across eight protein panels (cardiometabolic, cardiometabolic II, inflammation, inflammation II, neurology, neurology II, oncology, and oncology II) (**Table S4**). The protein levels were provided by preprocessing them into Normalized Protein eXpression (NPX) values. Details on sample selection, quality control, and pre-processing procedures for the Olink assay can be referred to in previous publications (1, 2).

**Genotype data**

Genotype data (version 3) was available from UKB, including 487,409 participants. The UK BiLEVE array and the UK Biobank axiom array were utilized to process the blood samples, and the following quality control, imputation, and other details were in the previous publication (3). We used software PLINK 2.0 (4) to perform the additional quality control and excluded single nucleotide polymorphisms (SNPs) with call rate < 95%, minor allele frequency < 1%, and deviation from the Hardy–Weinberg equilibrium with *P* < 1×10^-15^. In addition, we only included individuals that had White British ancestry and no more than ten putative third-degree relatives.

**Phenome-wide association analysis of pain-related proteins**

We used phenome-wide association analysis to explore the relationship of pain-related proteins with a wide range of phenotypes. According to the results of pathway enrichment and tissue expression analysis, we selected five categories to perform the phenome-wide analysis, including blood indicators, lung function, neuropsychiatric diseases, digestive diseases, and brain volumes. The blood indicators included two UKB categories: blood biochemistry indicators (ID 17518) and blood cell counts (ID 100081). According to the previous study (5), we subdivided the blood biochemistry into ‘liver function’, ‘renal function’, ‘endocrine’, ‘immunometabolism’, and ‘bone and joint’, and the blood cell counts into ‘white blood cell’, ‘red blood cell’, and ‘platelet’. Brain volume was assessed by T1 imaging, including cortical and subcortical regions’ volumes from two UKB categories, Freesurfer desikan white (ID 192) and Freesurfer ASEG (ID 190), respectively. In total, we included 59 blood indicators (30 blood biochemistry indicators and 29 blood cell counts), 9 lung function measures, 11 neuropsychiatric diseases, 18 digestive diseases, and 82 brain volume measures (66 cortical regions and 16 subcortical regions). The other details are provided in **Tables S29**-**S31**.

**References**

1. Elliott P, Peakman TC (2008): The UK Biobank sample handling and storage protocol for the collection, processing and archiving of human blood and urine. *Int J Epidemiol* 37:234-244.

2. Sun BB, Chiou J, Traylor M, Benner C, Hsu YH, Richardson TG, et al. (2023): Plasma proteomic associations with genetics and health in the UK Biobank. *Nature* 622:329-338.

3. Bycroft C, Freeman C, Petkova D, Band G, Elliott LT, Sharp K, et al. (2018): The UK Biobank resource with deep phenotyping and genomic data. *Nature* 562:203.

4. Purcell S, Neale B, Todd-Brown K, Thomas L, Ferreira MA, Bender D, et al. (2007): PLINK: a tool set for whole-genome association and population-based linkage analyses. *The American journal of human genetics* 81:559-575.

5. Wainberg M, Kloiber S, Diniz B, McIntyre RS, Felsky D, Tripathy SJ (2021): Clinical laboratory tests and five-year incidence of major depressive disorder: a prospective cohort study of 433,890 participants from the UK Biobank. *Transl Psychiatry* 11:380.

# Supplementary Figures


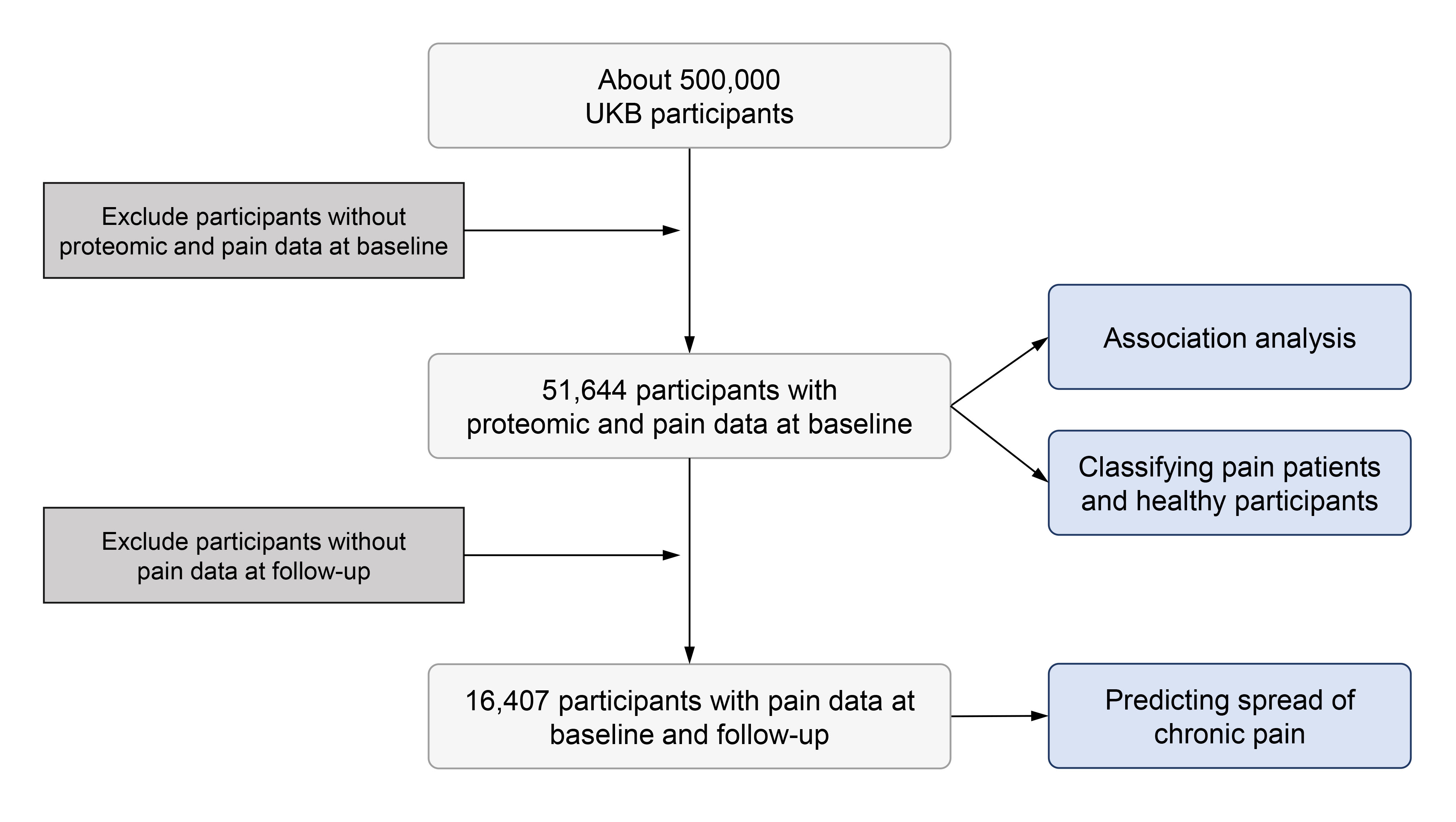


**Figure S1. The sample sizes and exclusion criteria used for analyses**


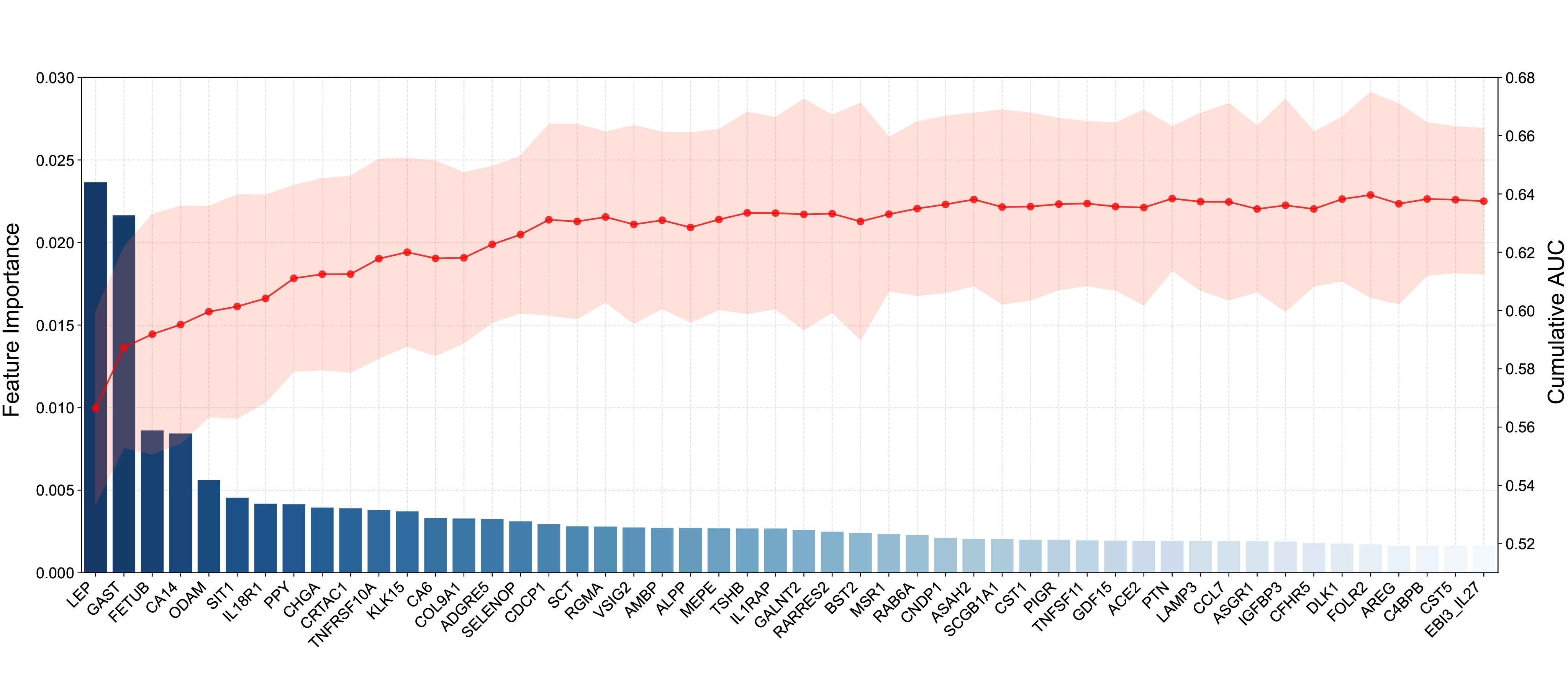


**Figure S2. The importance of features in the neck or shoulder pain model**

Bar chart shows the ranking of the importance of the variables according to their contribution to the model classification. Line chart shows the cumulative AUC value of the model that adds a feature in order at each iteration.


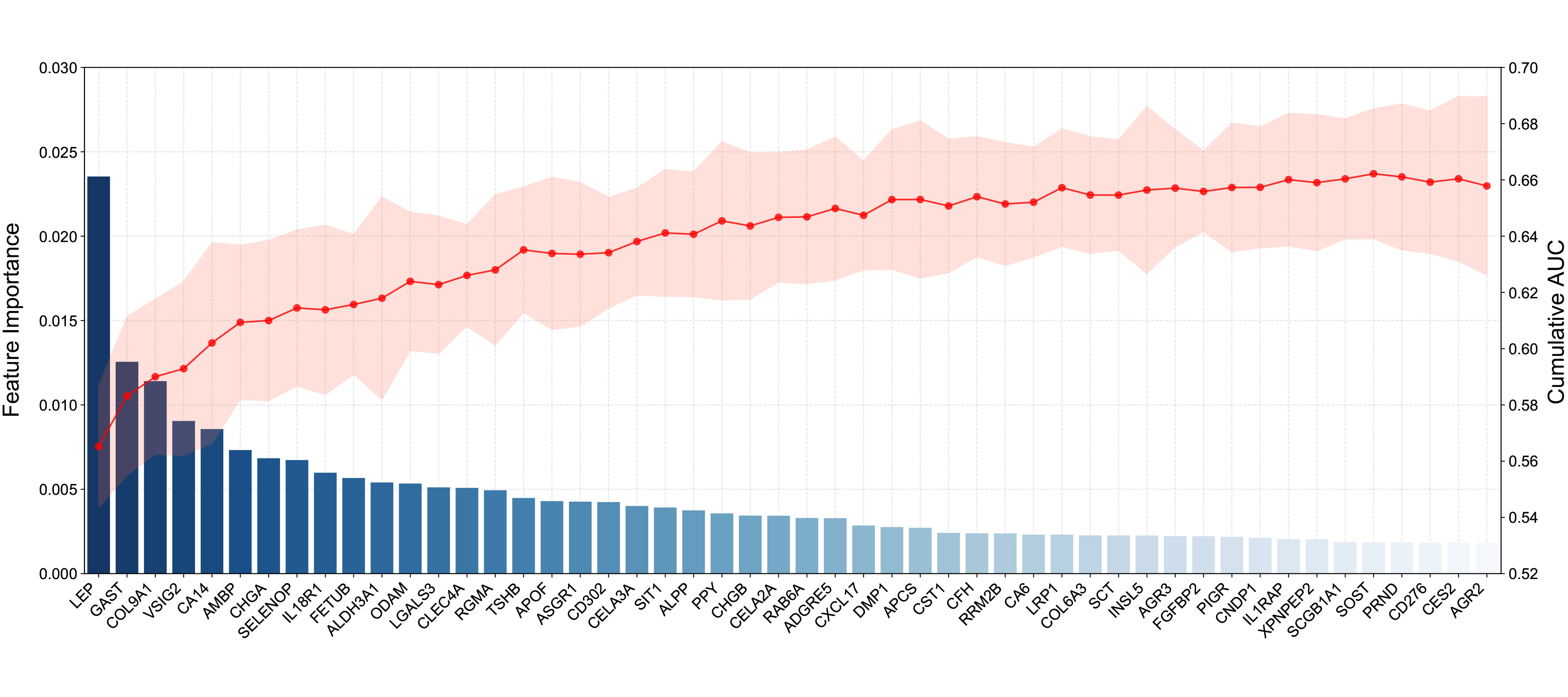


**Figure S3. The importance of features in the back pain model**

Bar chart shows the ranking of the importance of the variables according to their contribution to the model classification. Line chart shows the cumulative AUC value of the model that adds a feature in order at each iteration.


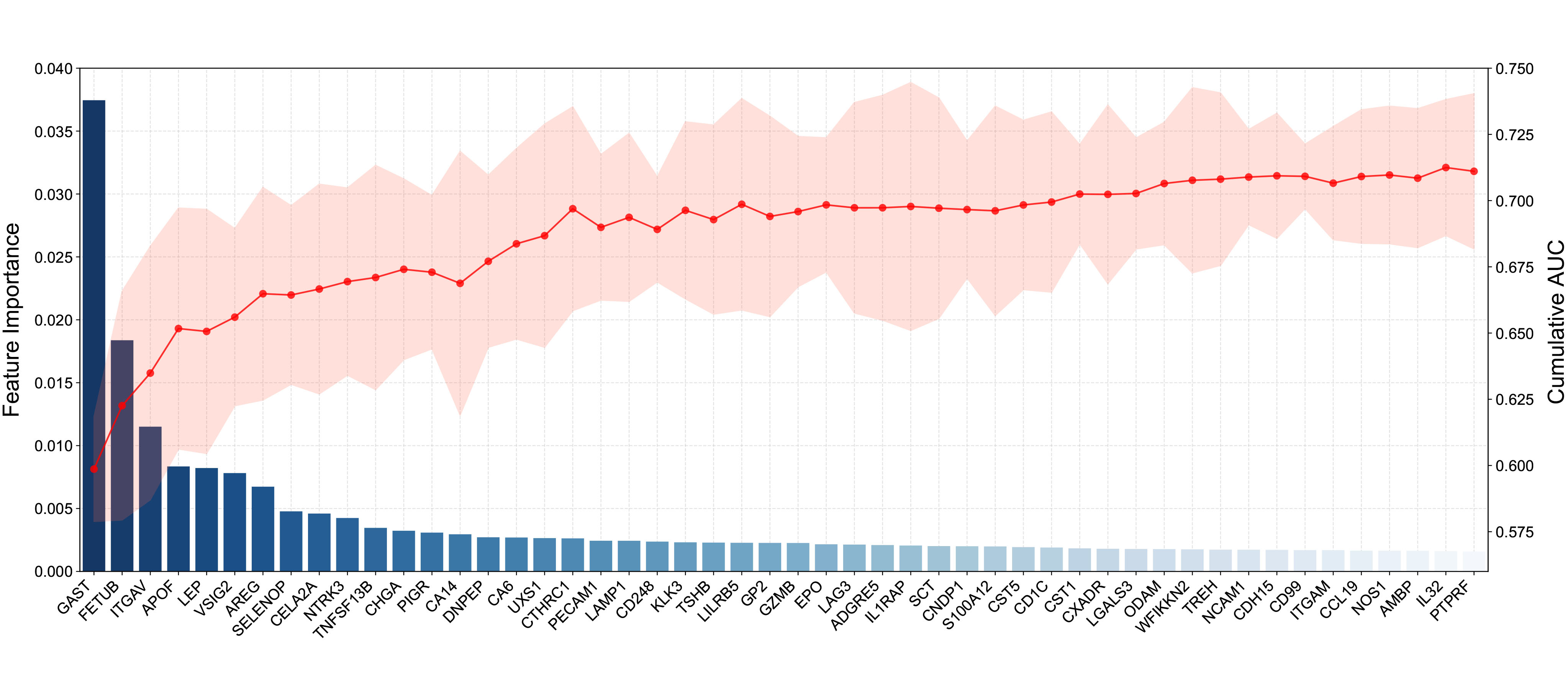


**Figure S4. The importance of features in the stomach or abdominal pain model**

Bar chart shows the ranking of the importance of the variables according to their contribution to the model classification. Line chart shows the cumulative AUC value of the model that adds a feature in order at each iteration.


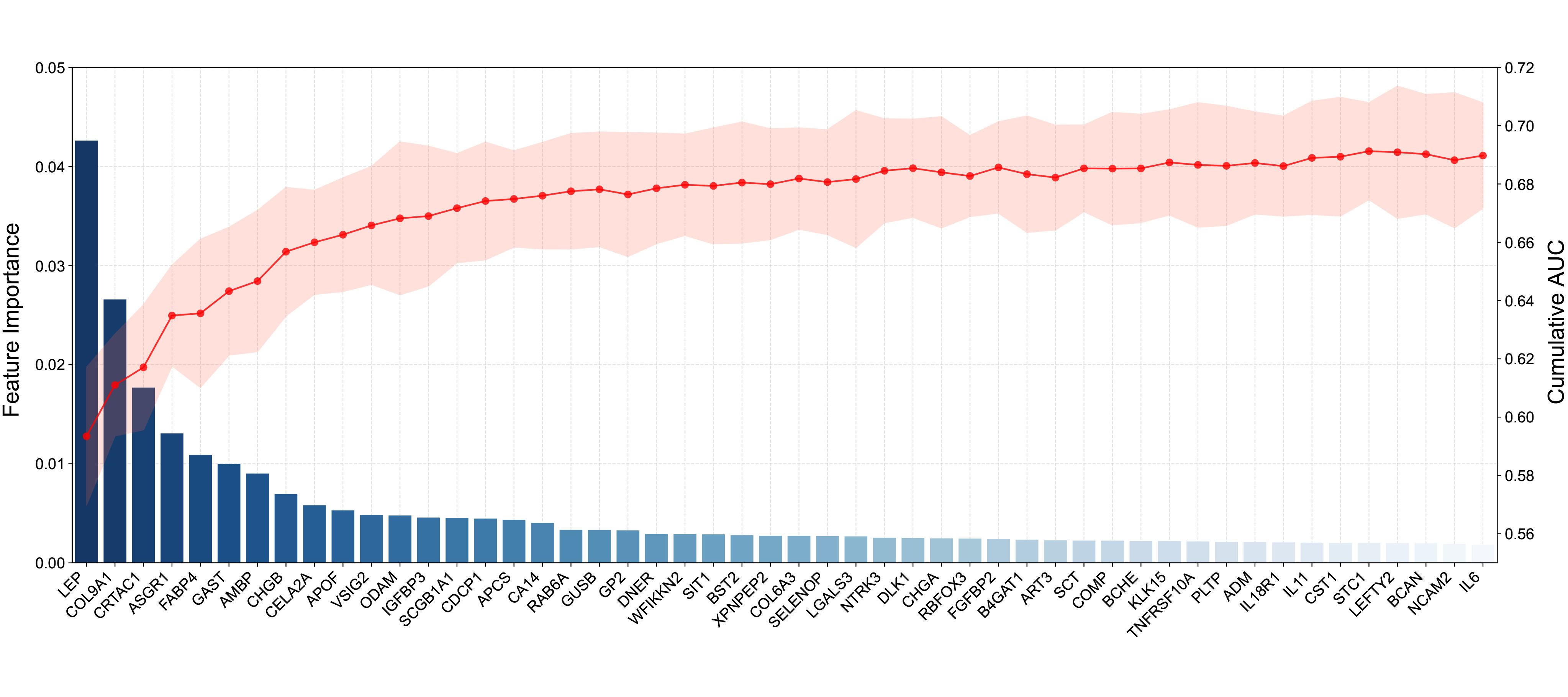


**Figure S5. The importance of features in the knee pain model**

Bar chart shows the ranking of the importance of the variables according to their contribution to the model classification. Line chart shows the cumulative AUC value of the model that adds a feature in order at each iteration.


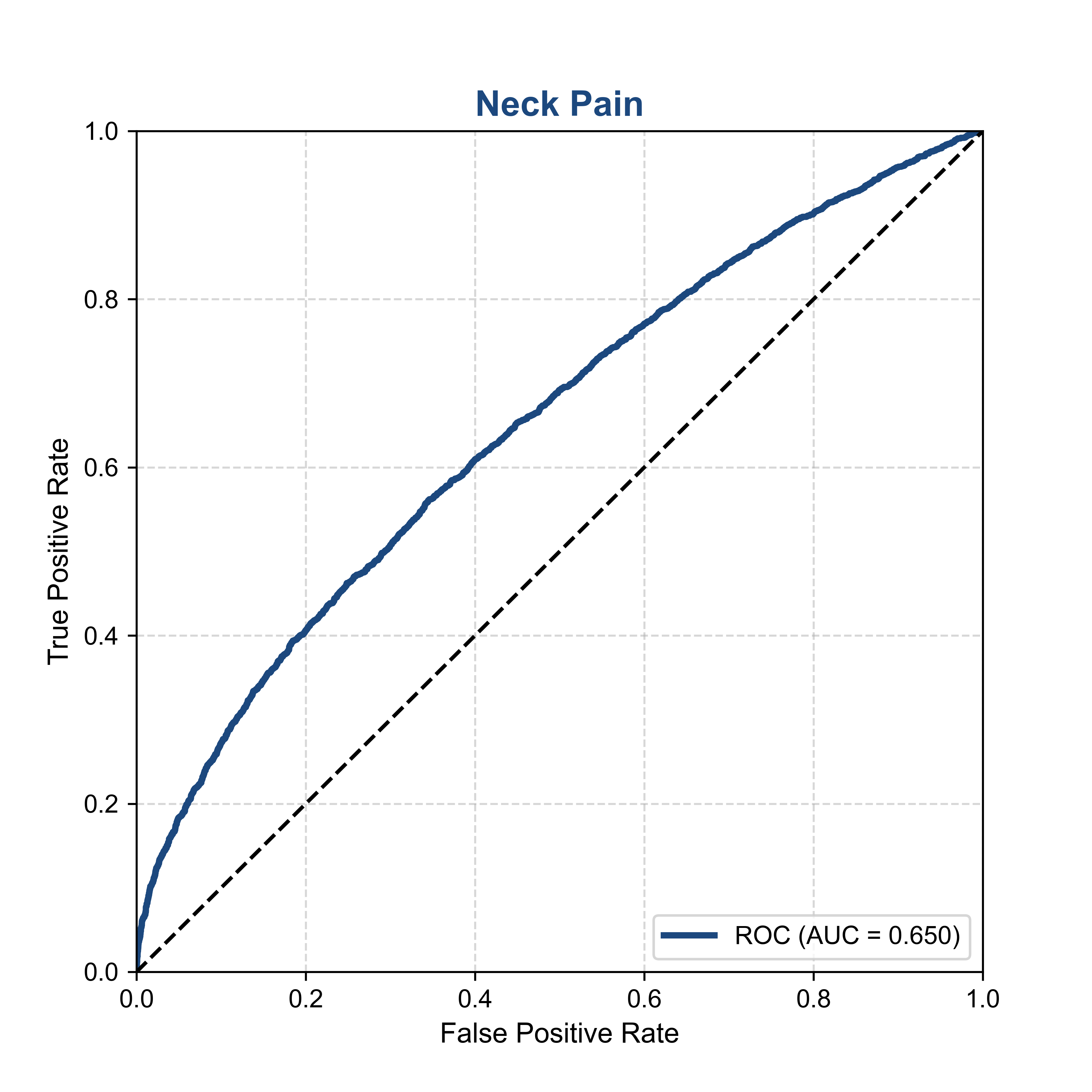


**Figure S6. ROC curve of the neck or shoulder pain model**

AUC indicates the area under the ROC curve. In the coordinate system, the vertical axis is TPR (true positive rate), and the maximum value is 1. The horizontal axis is FPR (false positive rate) and the maximum value is 1. The dashed line is the reference line (minimum standard), and the blue curve is the ROC curve.


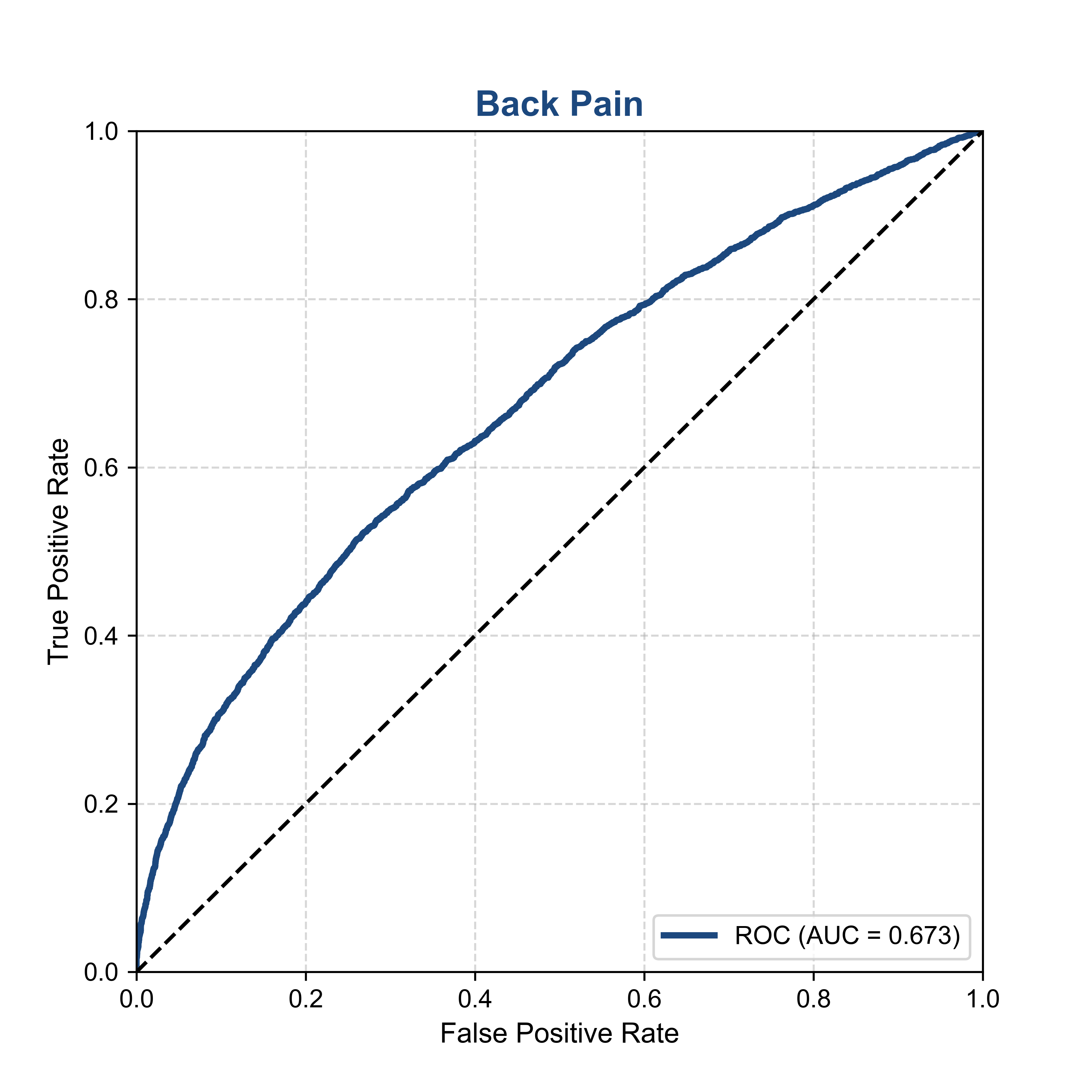


**Figure S7. ROC curve of the back pain model**

AUC indicates the area under the ROC curve. In the coordinate system, the vertical axis is TPR (true positive rate), and the maximum value is 1. The horizontal axis is FPR (false positive rate) and the maximum value is 1. The dashed line is the reference line (minimum standard), and the blue curve is the ROC curve.


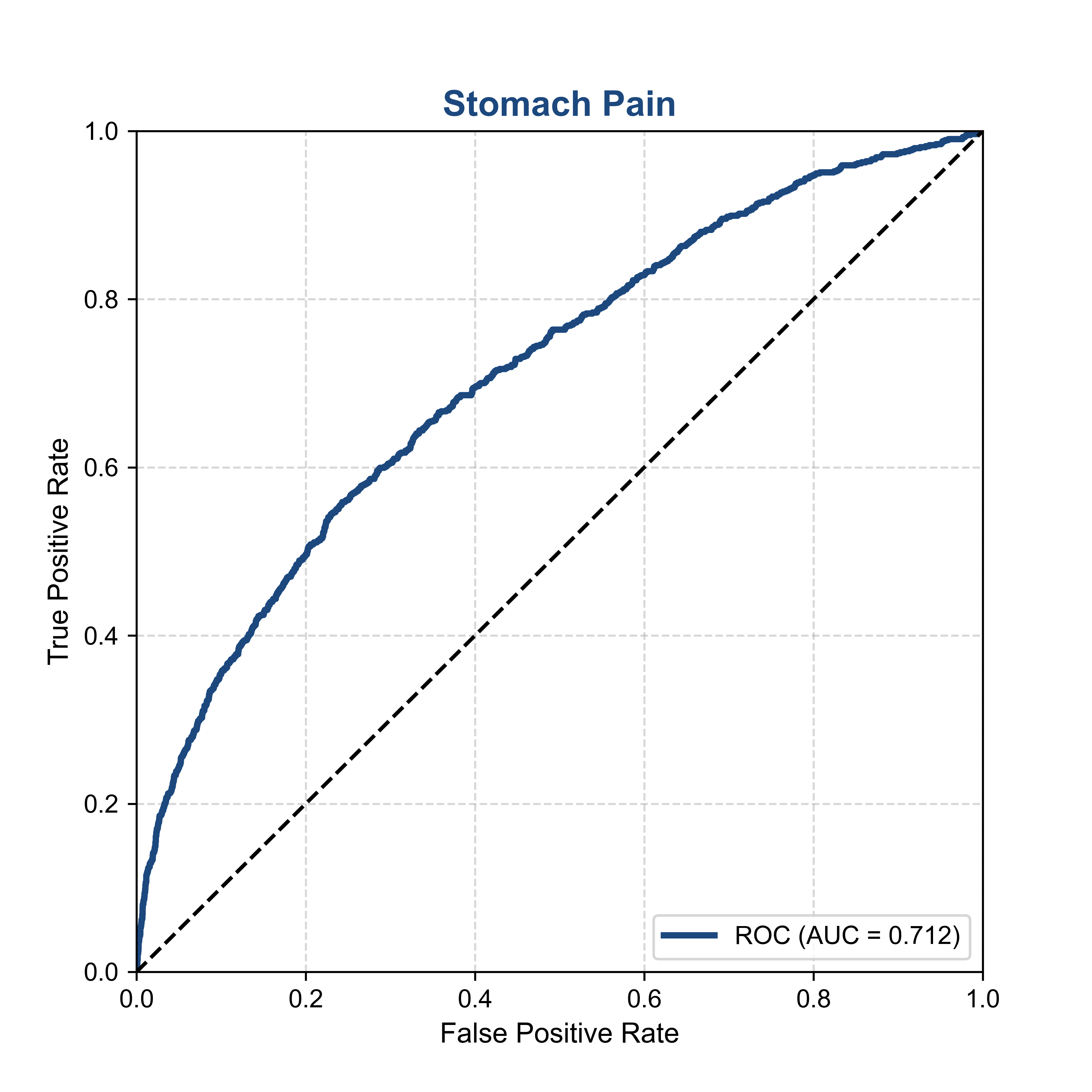


**Figure S8. ROC curve of the stomach or abdominal pain model**

AUC indicates the area under the ROC curve. In the coordinate system, the vertical axis is TPR (true positive rate), and the maximum value is 1. The horizontal axis is FPR (false positive rate) and the maximum value is 1. The dashed line is the reference line (minimum standard), and the blue curve is the ROC curve.


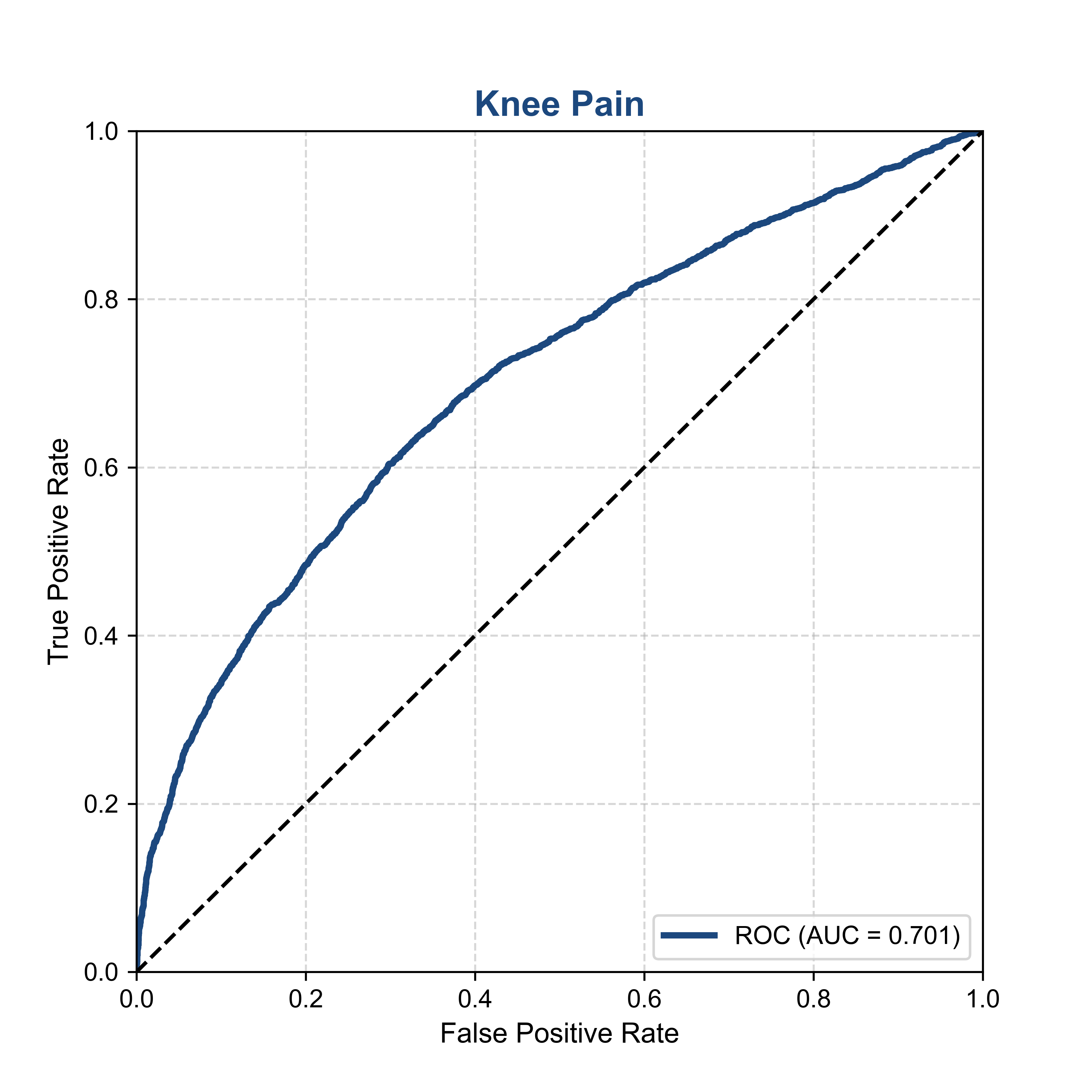


**Figure S9. ROC curve of the knee pain model**

AUC indicates the area under the ROC curve. In the coordinate system, the vertical axis is TPR (true positive rate), and the maximum value is 1. The horizontal axis is FPR (false positive rate) and the maximum value is 1. The dashed line is the reference line (minimum standard), and the blue curve is the ROC curve.
